# Impact of segmentation and discretization on radiomic features in ^68^Ga-DOTA-TOC PET/CT images of neuroendocrine tumor

**DOI:** 10.1186/s40658-021-00367-6

**Published:** 2021-02-27

**Authors:** Virginia Liberini, Bruno De Santi, Osvaldo Rampado, Elena Gallio, Beatrice Dionisi, Francesco Ceci, Giulia Polverari, Philippe Thuillier, Filippo Molinari, Désirée Deandreis

**Affiliations:** 1 Nuclear Medicine Unit, Department of Medical Sciences, University of Turin, Corso Dogliotti 14, 10126 Turin, Italy; 2 Biolab, Department of Electronics and Telecomunications, Politecnico di Torino, Turin, Italy; 3 Medical Physics Unit, AOU Città della Salute e della Scienza, Turin, Italy; 4 Department of Endocrinology, University Hospital of Brest, Politecnico di Torino Brest, Turin, France

**Keywords:** Texture analysis, Radiomics, Neuroendocrine tumor, Robustness, ^68^Ga-DOTATOC PET/CT, Semi-automatic segmentation

## Abstract

**Objective:**

To identify the impact of segmentation methods and intensity discretization on radiomic features (RFs) extraction from ^68^Ga-DOTA-TOC PET images in patients with neuroendocrine tumors.

**Methods:**

Forty-nine patients were retrospectively analyzed. Tumor contouring was performed manually by four different operators and with a semi-automatic edge-based segmentation (SAEB) algorithm. Three SUV_max_ fixed thresholds (20, 30, 40%) were applied. Fifty-one RFs were extracted applying two different intensity rescale factors for gray-level discretization: one absolute (AR60 = SUV from 0 to 60) and one relative (RR = min-max of the VOI SUV). Dice similarity coefficient (DSC) was calculated to quantify segmentation agreement between different segmentation methods. The impact of segmentation and discretization on RFs was assessed by intra-class correlation coefficients (ICC) and the coefficient of variance (COV^*L*^). The RFs’ correlation with volume and SUV_max_ was analyzed by calculating Pearson’s correlation coefficients.

**Results:**

DSC mean value was 0.75 ± 0.11 (0.45–0.92) between SAEB and operators and 0.78 ± 0.09 (0.36–0.97), among the four manual segmentations. The study showed high robustness (ICC > 0.9): (a) in 64.7% of RFs for segmentation methods using AR60, improved by applying SUV_max_ threshold of 40% (86.5%); (b) in 50.9% of RFs for different SUV_max_ thresholds using AR60; and (c) in 37% of RFs for discretization settings using different segmentation methods. Several RFs were not correlated with volume and SUV_max_.

**Conclusions:**

RFs robustness to manual segmentation resulted higher in NET ^68^Ga-DOTA-TOC images compared to ^18^F-FDG PET/CT images. Forty percent SUV_max_ thresholds yield superior RFs stability among operators, however leading to a possible loss of biological information. SAEB segmentation appears to be an optimal alternative to manual segmentation, but further validations are needed. Finally, discretization settings highly impacted on RFs robustness and should always be stated.

**Supplementary Information:**

The online version contains supplementary material available at 10.1186/s40658-021-00367-6.

## Introduction

Neuroendocrine tumors (NET) are a heterogeneous group of malignancies represented by different histological subtypes and different primary locations [1]. Histopathology is crucial in tumor classification and Ki-67 is currently used to define tumor grading in GEP NET [2]. However, the assessment of tumor aggressiveness is generally assessed by lesion biopsy, leading to a potential grading underestimation [3], since tumor heterogeneity is both spatial (inter- and intra-tumoral heterogeneity) and time-related (more aggressive cell clones developing over time) [4, 5]. Thus, although multiple-lesion biopsy sampling is not feasible, grading heterogeneity among primary and secondary lesions is not negligible [6]. New generation imaging technologies, including positron emission tomography (PET), might offer its contribution in the evaluation of tumor heterogeneity [7–9]. At present, PET imaging with ^68^Ga-DOTA-peptides analog to the somatostatin receptors (SSTR) is considered the state of the art to quantify SST receptors in vivo [10, 11], while ^18^F-fluorodeoxyglucose (^18^F-FDG) PET-CT is used to metabolically characterize more aggressive and higher grade NET lesions [12]. This dual approach has been recently evaluated leading to the development of the NETPET score [13]. Nevertheless, the simple in vivo quantification of receptor expression is not sufficient to characterize the biology of the tumor and the intra-patients and intra-tumor heterogeneity. This drawback might be solved with a better characterization of tumor heterogeneity by the extraction of radiomic features (RFs), as a surrogate biomarker for NET lesions characterization [14], from the ^68^Ga-DOTA-peptide PET-CT [15–18]. While scientific interest in radiomics applied to PET imaging is rapidly increasing, the methodological approach needs to be validated and standardized and, thus, harmonization among protocols is needed [19–21]. Indeed, imaging analysis procedures such as tumor segmentation methods, gray-level intensity discretization, and image reconstruction algorithm can affect the RFs [22–25]. Robustness analysis measures the variability of RFs concerning these factors. The identification of robust RFs for ^68^Ga-DOTA-TOC PET-CT is fundamental since this innovative modality might be used as a prognostic and predictive tool for evaluating tumor heterogeneity in NET. To our knowledge, there is only one study evaluating the robustness of RFs in function of image acquisition and reconstruction parameters for ^68^Ga-DOTA-peptides PET/CT (without considering the consequences of different segmentation approaches) [26], while the extraction of RFs and the assessment of RFs robustness in ^18^F-FDG PET/CT imaging has been broadly explored [27–31]. There are several reasons to evaluate the RFs robustness specifically in ^68^Ga-DOTA-peptide tracers: a diverse range of positrons compromising the resolution in PET in a different way comparing to ^18^F-FDG [32–34]; a different physiological distribution of ^68^Ga-DOTA-peptide; and a high inter-patient and intra-patient heterogeneity for both physiological and pathological uptake comparing to ^18^F-FDG [35], leading to the necessity to provide different segmentation methods and discretization settings.

The objective of this study was to evaluate the robustness of RFs in function of segmentation methods and discretization settings in ^68^Ga-DOTATOC PET/CT images.

## Materials and methods

### Patient selection

270 consecutive patients affected by NET referred to our institution to perform ^68^Ga-DOTA-TOC PET/CT between February 2017 and July 2019 were reviewed (IRB protocol: CS2/477). The inclusion criteria of the present study were (1) histologically proven NET, (2) patients who underwent ^68^Ga-DOTA-TOC PET/CT for staging in treatment-naïve patients or restaging after surgery, and (3) willing to sign an informed consent form (ICF). Exclusion criteria were (1) age < 18 years and (2) previous systemic therapies (e.g., somatostatin analogs, chemotherapy, everolimus, and peptide receptor radionuclide therapy). Forty-nine patients with a total of 60 lesions matched the inclusion criteria and were considered in this analysis. Primary tumor sites were GEP-NET, lung NET, and others NET in 77.5% (38/49), 18.4% (9/49), and 4.1% (2/49) of cases, respectively. Patients’ characteristics are exposed in detail in Table 1.

**Table 1:** Demographic data and NET characteristics of study subjects (values are given as mean ± standard deviation and range)

Demographic data and tumor characteristics			
Characteristic		***n***	%
**Number of patients**		49	100%
**Sex**	Female	22	44.9%
	Male	27	55.1%
**Age, years** **mean ± sd (range)**		61.7 ± 14.1 (18–83)	
**Weight, kg** **mean ± sd (range)**		76.2 ± 17.1 (48–115)	
**Injected tracer activity, MBq** **mean ± sd (range)**		145.1 ± 25.2 (100–212)	
**Primary**	**GEP-NET total**	38	77.5%
	Pancreatic	22	57.9%
	Gastro-enteric	16	42.1%
	**Lung**	9	18.4%
	**Other**	2	4.1%
**NET histological sub-types**	G1	33	67.4%
	G2	6	12.2%
	G3	1	2.0%
	Atypical carcinoid	4	8.2%
	Typical carcinoid	5	10.2%
**KI67, %** **mean ± sd (range)**		4.2 ± 12.9 (1–90)	
**Number of lesions**	Primary	42	70.0%
	Liver metastasis	8	13.3%
	Bone metastasis	0	0%
	Lymph node metastasis	9	15.0%
	Other metastasis	1	1.7%

### PET/CT acquisition and image reconstruction

All patients underwent PET/CT on an analog 3-dimensional (3D) PET scanner (Philips Gemini Dual-slice EXP scanner—PET Allegro^TM^ system with Brilliance CT scanner—Philips Medical Systems, Cleveland, OH). In accordance with the procedural guidelines for PET imaging [36–38], the injected tracer activity was 145.1 ± 25.3 MBq of ^68^Ga-DOTA-TOC (range, 100–212 MBq). After 60 min of uptake and following free-breathing CT acquisition for attenuation correction from the vertex of the skull to the mid-thighs (5 mm slice, 40 mAs, and 120 kVp), PET data were acquired in 3-dimensional (3D) mode, covering the same anatomical region of the CT, with 2.5 min per bed position and 6–8 bed positions per patient. The PET scans were reconstructed by ordered subset expectation maximization (OSEM) algorithm (3D-RAMLA), with the following settings: 4 iterations, 8 subsets, and field of view (FOV) of 576 mm. For all reconstructions, matrix size was 144 × 144 voxels, resulting in isotropic voxels of 4.0 × 4.0 × 4.0 mm^3^. All acquisitions were corrected for photon attenuation (using the corresponding CT image), as well as for scatter and random coincidences.

### Lesion segmentation

For each lesion, a three-dimensional volume of interest (VOI) was manually delineated (VOI_*m*_), slice-by-slice, in the OSEM PET images by four independent observers, all nuclear medicine physicians (FC, VL, GP, and BD with 10, 7, 5, and 3 years of expertise, respectively), by using the software LifeX v. 4.81 (IMIV/CEA, Orsay, France—www.lifexsoft.org) [39].

**Fig. 1:**
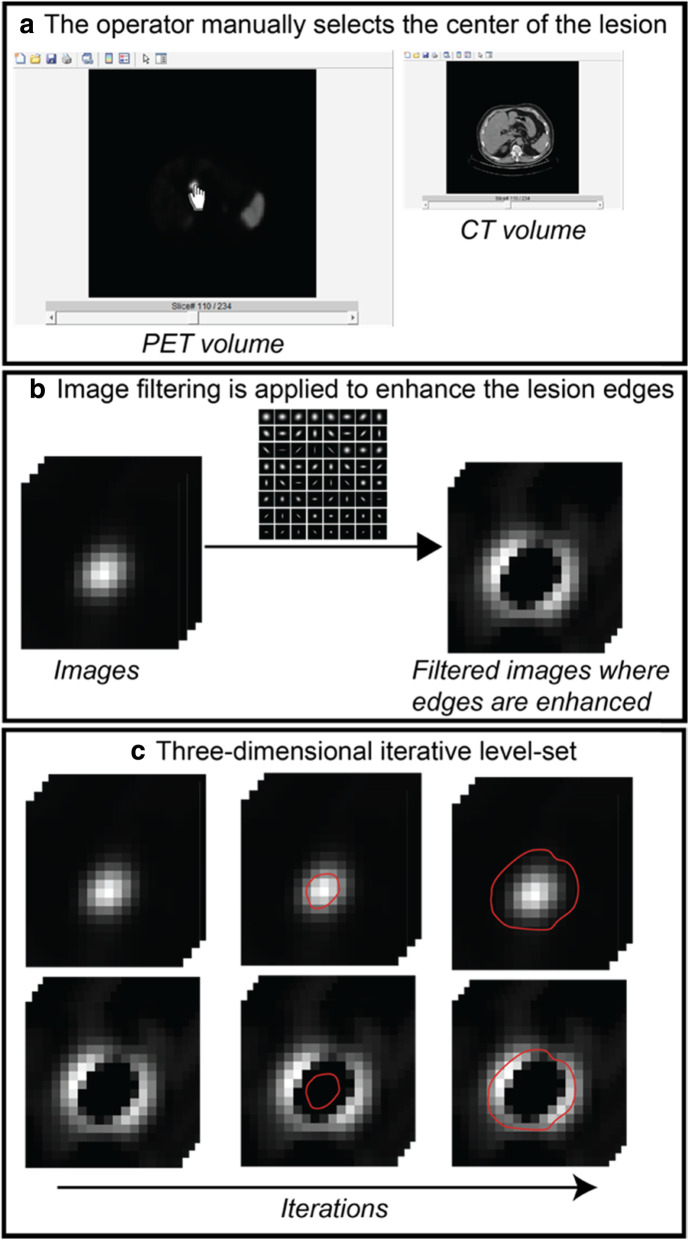
Semi-automatic edge-based (SAEB) algorithm workflow. The algorithm requires the intervention of an operator to insert the central point of the lesion (**a**) through an interface created in MATLAB, the operator can view both the PET and the CT images separately. As a second step, edge-preserving filters are applied to the image to emphasize the edge of the lesion (**b**) and, after that, and a level-set is used which is a shape that evolves iteratively over the image (**c**). The level-set acts both on the original image of the lesion and on the filtered image in which the contours are highlighted. The iteration 0 of the level set, which would be the initialization, is the center of the lesion indicated by the operator. The final outline of the lesion is achieved at the end of the process

In additional, each lesion was also contoured using a semi-automatic edge-based (SAEB) algorithm (VOI_SAEB_), homemade implemented in MATLAB (MathWorks) code, based on the active contour model proposed by Chan and Vese [40]. The algorithm is semi-automatic since the operator intervention was required in order to insert the central point of the lesion (Fig. 1a). The developed MATLAB graphical user interface allowed the operator to view both the PET and the CT images separately. Edge enhancement filters were applied to emphasize the edges of the lesion (Fig. 1b) and, subsequently, a curve was evolved iteratively on both the original and the edge-enhanced image in order to match the lesion contours by using a level-set formulation (Fig. 1c). The iteration 0 of the level-set, which is the initialization, was the center of the lesion indicated by the operator. The final contour of the lesion (VOI_SAEB_) was achieved at the end of the iterative level-set.

A threshold-based segmentation approach was implemented applying three different thresholds on both manual VOI_*m*_ and VOI_SAEB_, defined as 20, 30, and 40% of the SUV_max_ (VOI_20_, VOI_30_, and VOI_40_, respectively), as recently suggested by Toriihara et al. [41]. This approach is different from the well-established isocontouring methods frequently used for ^18^F-FDG PET/CT, in which the threshold is applied to larger regions of interest containing the tumor [42, 43]. In ^68^Ga-DOTA-peptide tracers, the methodology proposed by Toriihara is preferable to exclude surrounding physiological uptake, especially for the segmentation of liver metastases which are very prevalent in metastatic neuroendocrine tumors.

### Intensity discretization

To perform RFs calculation, in particular of textural features, voxels values were redefined considering a limited number of SUV intensity values (gray-level intensity discretization process). To investigate the effect of gray-level discretization, the analysis was performed with two different settings of intensity discretization:

- Absolute resampling with 64 number of gray levels (bins) between 0 and 60 SUV units (“fixed bin size” equal to 0.95, using the IBSI nomenclature [44]), called AR60, since most of the lesions presented a SUV_max_ between 0 and 60, as showed in supplemental material (Figure S1A);
- Relative resampling (RR), between minimum and-maximum SUV of the VOI, using a “fixed bin number” equal to 64 number of gray levels (using the IBSI nomenclature [44]) and different sizes of bin, according to the uptake characteristic of each lesion/VOI.

Number of gray levels was set to 64 based on the results of previous studies regarding RFs robustness in ^18^F-FDG PET/CT [45–47]. The spatial resampling was of 4 × 4 × 4 mm^3^, according to the resolution limits of the Philips Gemini Dual-slice EXP PET/CT scanner.

### Radiomic features extraction

**Fig. 2:**
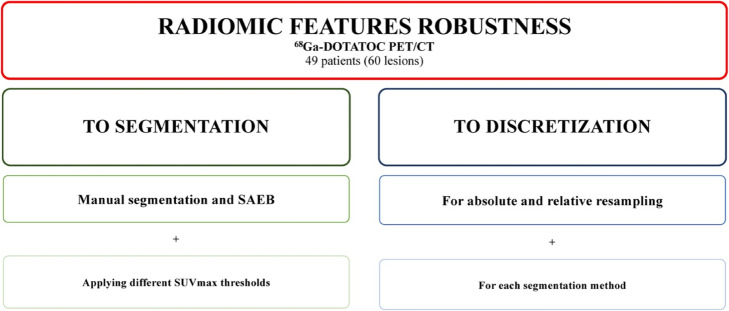
Flow chart of the ICC and COV^*L*^ analysis applied to assess radiomic features robustness according to segmentation and discretization

Radiomics features were extracted from PET images in all the VOI_*m*_ segmented by each nuclear medicine physician, in all the VOI_SAEB_ and in all the corresponding VOI_20_, VOI_30_, and VOI_40_ using the two intensity rescaling factors (AR60 and RR). Hence, a total of 40 combinations of VOI, threshold, and intensity rescaling factors were tested, as shown in Fig. 2.

A total of fifty-one semi-quantitative PET parameters and RFs were extracted, using LifeX description:

- Ten conventional PET parameters: such as SUV_max_, SUV_mean_, SUV_min_, SUV_peak_, SUV_std_ SUV quartiles (SUVQ1, SUVQ2, and SUVQ3) and total lesion somatostatin receptor expression (TLSRE);
- Five descriptors of the image intensity histogram: HISTO_Skewness (asymmetry), HISTO_Kurtosis (flatness), HISTO_Energy (uniformity), HISTO_Entropy_log2, and _log10 (randomness);
- Four shape-based features, that describe shape and size of VOI: SHAPE_Volume (mL), SHAPE_Volume (voxels), SHAPE_Sphericity, and SHAPE_Compacity; and
- Thirty-two textural features: (a) seven features from gray-level co-occurrence matrix (GLCM): describing the correlation between pair of voxels in 13 directions of a three-dimensional space; (b) eleven features from gray-level run length matrix (GLRLM): describing the number and length of run with a certain level of gray in 13 directions of a three-dimensional space; (c) eleven features from gray-level zone length matrix (GLZLM): describing the number and size of zone with a certain level of gray in 13 directions of a three-dimensional space; (d) three features from neighborhood gray-level different matrix (NGLDM): describing the difference between a voxel and its connected neighbors.

Detailed descriptions of these features can be found in the LifeX documentation (www.lifexsoft.org) [39]. Supplementary Table 1 reports the complete list of computed RFs and the comparison between LifeX and the Imaging Biomarker Standardization Initiative (IBSI) nomenclatures [44]. The GLZLM features of LifeX correspond to gray-level size zone matrix (GLSZM) of IBSI; the two features categories gray-level distance zone matrix (GLDZM) and neighboring gray tone difference matrix (NGTDM) are not measurable in LifeX.

### Statistical analyses

Quantitative comparisons between VOI_*m*_ and VOI_SAEB_ were evaluated through the Dice similarity coefficient (DSC), which measures spatial overlap between two different segmentations of the same lesion:

$$ \mathrm{DSC}\left({V}_1,{V}_2\right)=2\frac{\left|{V}_1\cap {V}_2\right|}{\left|{V}_1\right|+\left|{V}_2\right|} $$

where |*V*_1_| and |*V*_2_| were the volumes of the two segmentations to be compared, |*V*_1_ ∩ *V*_2_| was the volume of the overlap between *V*_1_ and *V*_2_. DSC values can range from 0, when the two segmentations have no overlap, to 1 when the two segmentations are coincident [42].

The algorithm for simultaneous truth and performance level estimation (STAPLE), which takes a collection of segmentations of an image and computes a probabilistic estimate of the true segmentation [48], was also used to compare VOI_SAEB_ with the “true” segmentation (VOI_STAPLE_) derived by the four VOI_*m*_.

Robustness of RFs was assessed by two-way mixed effects intra-class correlation coefficients (ICC) to evaluate consistency and coefficient of variance for each lesion (COV^*L*^) to evaluate agreement in the various settings.

The intra-class correlation coefficients (ICC) was defined as:

$$ \mathrm{ICC}=\frac{\mathrm{BMS}-\mathrm{RMS}}{\mathrm{BMS}+\left(N-1\right)\times \mathrm{RMS}} $$

where BMS is the between-subjects mean square, RMS is the residual mean square and *N* is the number of measurements of the RF (e.g., 2 in the case of intensity discretization, being AR60 and RR). RFs were considered highly robust in case of ICC > 0.9, robust if ICC > 0.8, moderately robust if ICC was between 0.5 and 0.8 and poorly robust if ICC was < 0.5 [25, 49, 50].

The coefficient of variation (COV^*L*^), which is commonly used to measure relative dispersion, calculated for each lesion (*L*) was defined as:

$$ {\mathrm{COV}}^L=100\times \frac{\sqrt{\frac{1}{N-1}\ {\sum}_{k=1}^N{\left({m}_k^L-{\underset{\_}{m}}^L\right)}^2}}{{\underset{\_}{m}}^L} $$

where ${m}_k^L$ is the measurement of RFs for lesion *L* for a specific segmentation and intensity discretization (k) and ${\underset{\_}{m}}^L$ is the mean value of lesion *L* over the considered combinations of segmentation and discretization approaches, as presented in the study of Bailly et al. [26]. RFs with low percentage of COV^*L*^ are characterized by low dispersion, on contrary RFs with high percentage of COV^*L*^ are characterized by high dispersion.

To investigate the relationship between RFs and lesion volume and SUV_max_, a Pearson’s correlation analysis was carried out. To consider a unique RF value for every segmentation, RFs values were averaged across segmentations.

All analyses were performed using statistical R software (R Foundation, Vienna, Austria [51]).

## Results

### Quantitative comparison of manual and SAEB segmentation

**Fig. 3:**
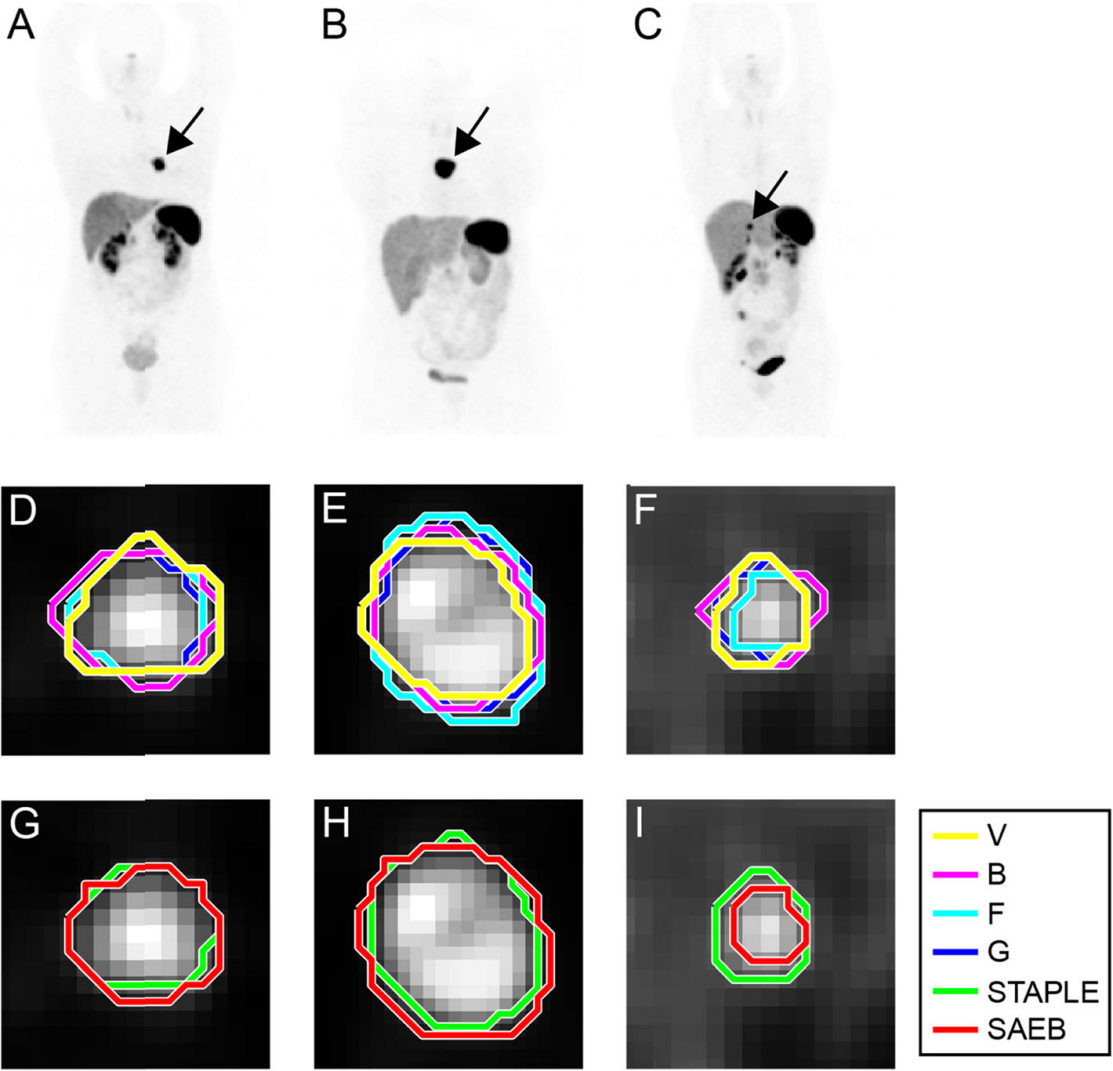
Example of segmentation (single slice images) of lesions extracted from three different examined patients. Panels **a**, **d**, and **g** show a lung primary NET; panels **b**, **e**, and **h** show metastatic lesion in a mediastinal lymph node; panels **c**, **f**, and **i** show a metastatic liver lesion. The first row shows the maximum intensity projection (MIP) of ^68^Ga-DOTATOC of the three different patients where black arrows point at the segmented lesions. In the second row, manual contours performed by the four operators are represented in different colors. The third row shows the STAPLE contour (in green) and the semi-automatic edge-based segmentation (SAEB) algorithm result (in red)

**Fig. 4:**
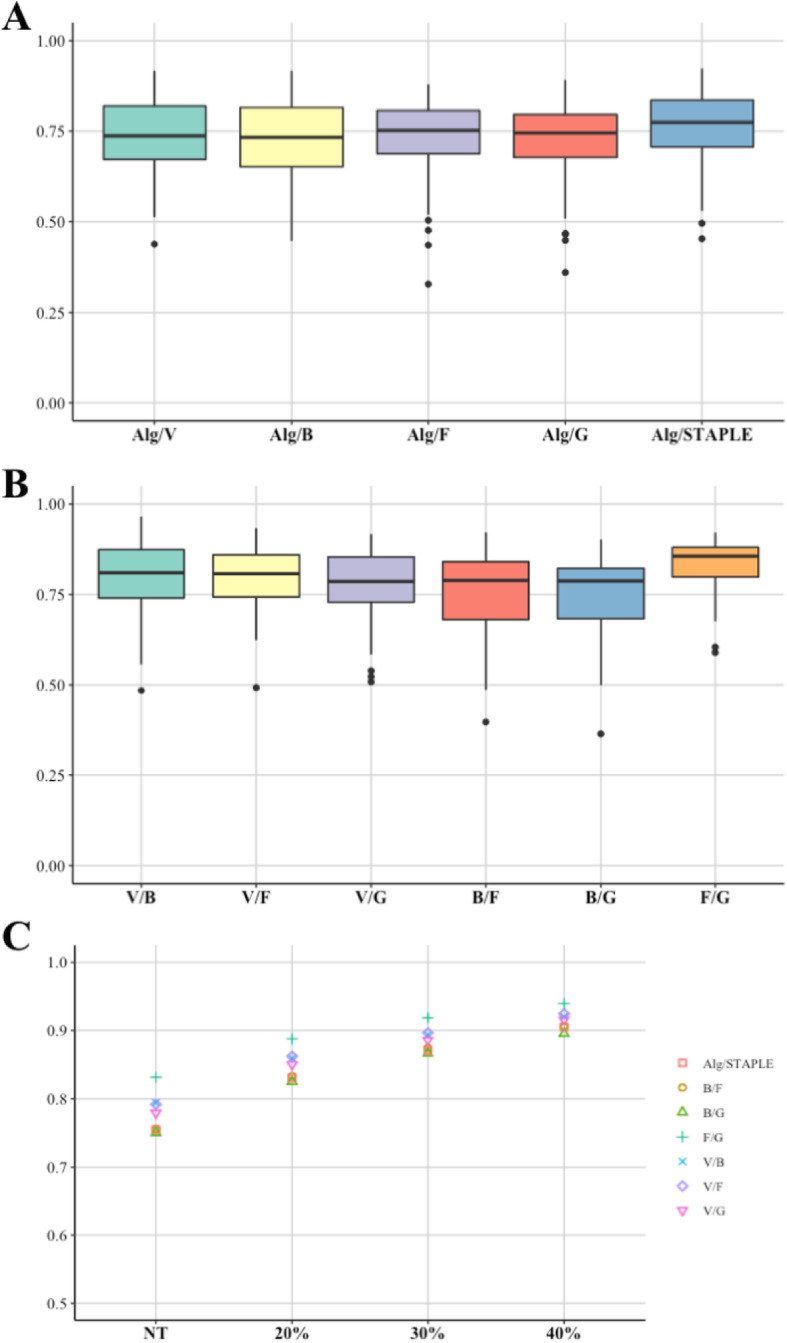
Boxplots of mean DSC comparing the semi-automatic edge-based algorithm (Alg) with manual segmentations and with STAPLE (**a**), comparing manual segmentations by different operators (**b**) and comparing different manual segmentations and Alg with STAPLE for different SUV_max_ thresholds (NT, no threshold applied) (**c**)

Mean value of DSC comparing VOI_SAEB_ with VOI_STAPLE_ was 0.75 ± 0.11 (0.45–0.92), while mean value of DSC among VOI_*m*_ was 0.78 ± 0.03 (0.75–0.83). Examples of segmentation performed by SAEB and operators for three different lesions are shown in Fig. 3. DSC boxplots between VOI_SAEB_ and the different VOI_*m*_ and between manual operators for each lesion are reported in Fig. 4a, b.

Comparison between operators showed a perfect segmentation overlap (DSC = 1) for 24 out of 60 lesions, applying the 40% SUV_max_ threshold; mean DSC using different SUV_max_ threshold are reported in Fig. 4c.

Mean DSC values improved as the SUV_max_ threshold applied increases. The volume distributions for different thresholds are shown in supplementary material (Figure S1B).

### Impact of different segmentation approaches on RF

**Fig. 5:**
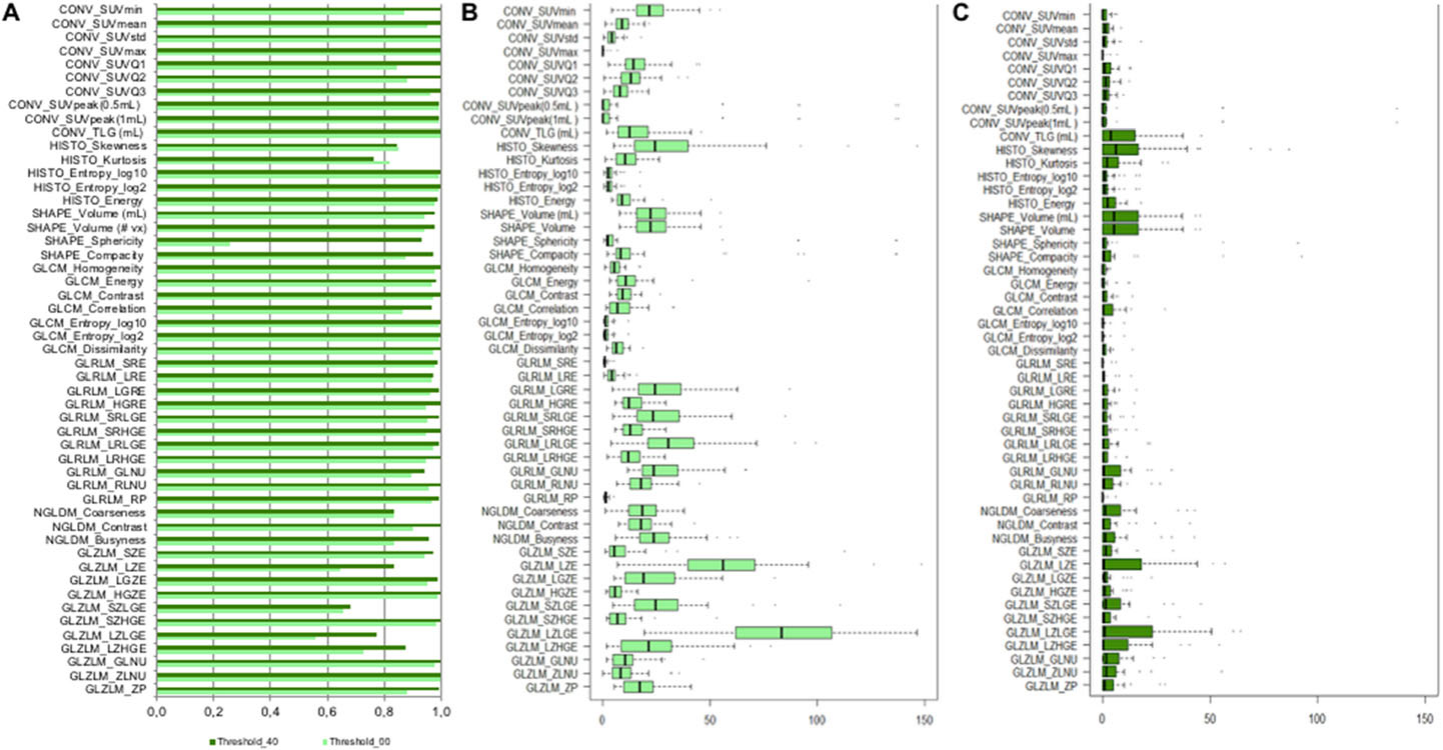
**a** Bar diagrams of intra-class correlation coefficient (ICC) values of RFs robustness to segmentation (different operators and semi-automatic algorithm), using AR60 and applying different SUV_max_ thresholds (no threshold and 40% SUV_max_ threshold applied). **b** Boxplot of percentage COV^*L*^ for segmentation (for the first operator, results similar for the other operators) for each RFs, using AR60 and applying no SUV_max_ threshold. **c** Boxplot of percentage COV^*L*^ applying 40% SUV_max_ threshold. TLG (total lesion glycolysis) conventional parameter in our study corresponded to the TLSRE (total lesion somatostatin receptor expression)

Using no threshold and applying AR60 intensity rescale factors, 64.7% of RFs showed high robustness (ICC > 0.9) to segmentation (7/10 conventional, 3/6 histogram, 2/4 shape, and 21/31 textural) (Fig. 5a).

Using a 40% SUV_max_ threshold, the robustness of RFs to segmentation (ICC > 0.9) increased to 86.5% of RFs. An increase of the SUV_max_ threshold produced a substantial increase of ICC of the following features: CONV_SUVmin, CONV_SUVQ1, CONV_SUVQ2, SHAPE_Sphericity, SHAPE_Compacity, GLCM_Correlation, NGLDM_Contrast, NGLDM_Busyness, GLZLM_LZE, GLZLM_LZLGE, GLZLM_LZHGE, and GLZLM_ZP while lower increase was observed for the rest of features (Fig. 5a). Further, using a 40% SUV_max_ threshold, textural features were not computed in 22 lesions due to low number of voxels.

About the corresponding COV^*L*^ analysis, when no threshold was applied, the grade of dispersion of the majority of RFs was rather low: median COV^*L*^ values were below 10% for 47% of RFs and below 20% for 75% of RFs (Fig. 5b). Only two RFs (GLZLM_LZE and GLZLM_LZLGE) showed a COV^*L*^ > 50%. Using a 40% SUV_max_ threshold, median values of COV^*L*^ were lower than 10% for all the RFs (Fig. 5c).

ICC and COV^*L*^ analysis regarding robustness to SUV_max_ thresholds (no threshold, 20%, 30%, 40%) using AR60 are shown in the supplemental material (Figure S2). 50.9% of RFs (5/10 conventional, 3/6 histogram, 0/4 shape and 18/31 textural) showed high robustness (ICC > 0.9). The results of COV^*L*^ showed a high variability of the majority of RFs in function of different SUV_max_ thresholds. Median value of COV^*L*^ was < 10% for few RFs, namely SUV_max_, SUVpeak (0.5 ml and 1 ml), HISTO_Entropy (log10 and log2), GLCM_Homogeneity, GLCM_Contrast, GLCM_Entropy (log10 and log2), GLCM_Dissimilarity, GLRLM_SRE, GLRLM_LRE, GLRLM_RP, GLZLM_SZE, and GLZM_ZP.

### Impact of different discretization settings on RFs

**Fig. 6:**
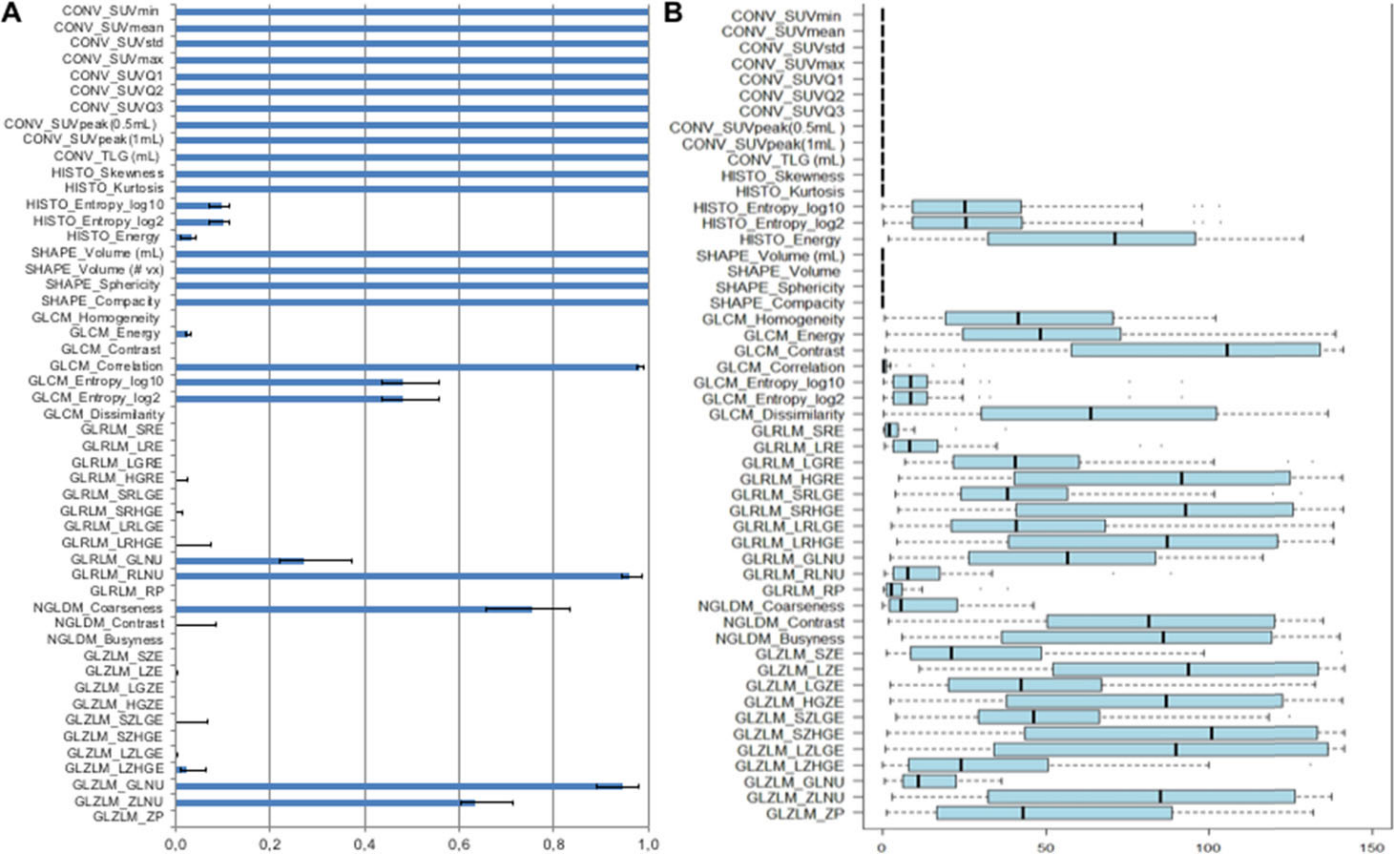
**a** Bar diagrams of intra-class correlation coefficient (ICC) values of RFs robustness to different intensity rescale factors (RR and AR60) when no threshold was applied for different operators and semi-automatic algorithm. Bars in blue show the median ICC between different segmentations, applying no threshold. Range error bars (in black) encompass the lowest and highest values for different operators. **b** Boxplot of COV^*L*^ for different intensity rescale factors (RR and AR60), applying no threshold, for the first operator (results superposable for the other operators). TLG (total lesion glycolysis) conventional parameter in our study corresponds to the TLSRE (total lesion somatostatin receptor expression)

Comparing the five VOI delineations (4 VOI_*m*_ and VOI_SAEB_) and applying no SUV_max_ threshold (Fig. 6a), median value of ICC for the two intensity discretization approaches (AR60 and RR) was equal to 1 for all conventional and shape features, HISTO_Skewness and HISTO_Kurtosis (not affected by the discretization), and for only three textural features, namely GLCM_Correlation, GLRLM_RLNU, and GLZLM_GLNU. Overall, the percentage of highly robust features was 37% (10/10 conventional, 2/5 histogram, 4/4 shape, and 3/32 textural). The majority of the remaining textural features showed very poor robustness to discretization settings except for NGLDM_Coarseness which had a median ICC > 0.7. The COV^*L*^ analysis (Fig. 6b) highlighted in general low COV^*L*^ values for all the RFs with high ICC (conventional, shape, HISTO_Skewness and HISTO_Kurtosis, GLCM_Correlation, GLRLM_RLNU, and GLZLM_GLNU). The majority of the other textural features were characterized by a very high dispersion, corresponding to a high percentage of COV^*L*^ value. Only GLCM_Entropy (log10 and log2), GLRLM_SRE, GLRLM_LRE, GLRLM_RP presented a COV^*L*^ < 10%, despite a corresponding low ICC for these RFs.

### RFs correlation with SUV_max_ and volume

**Fig. 7:**
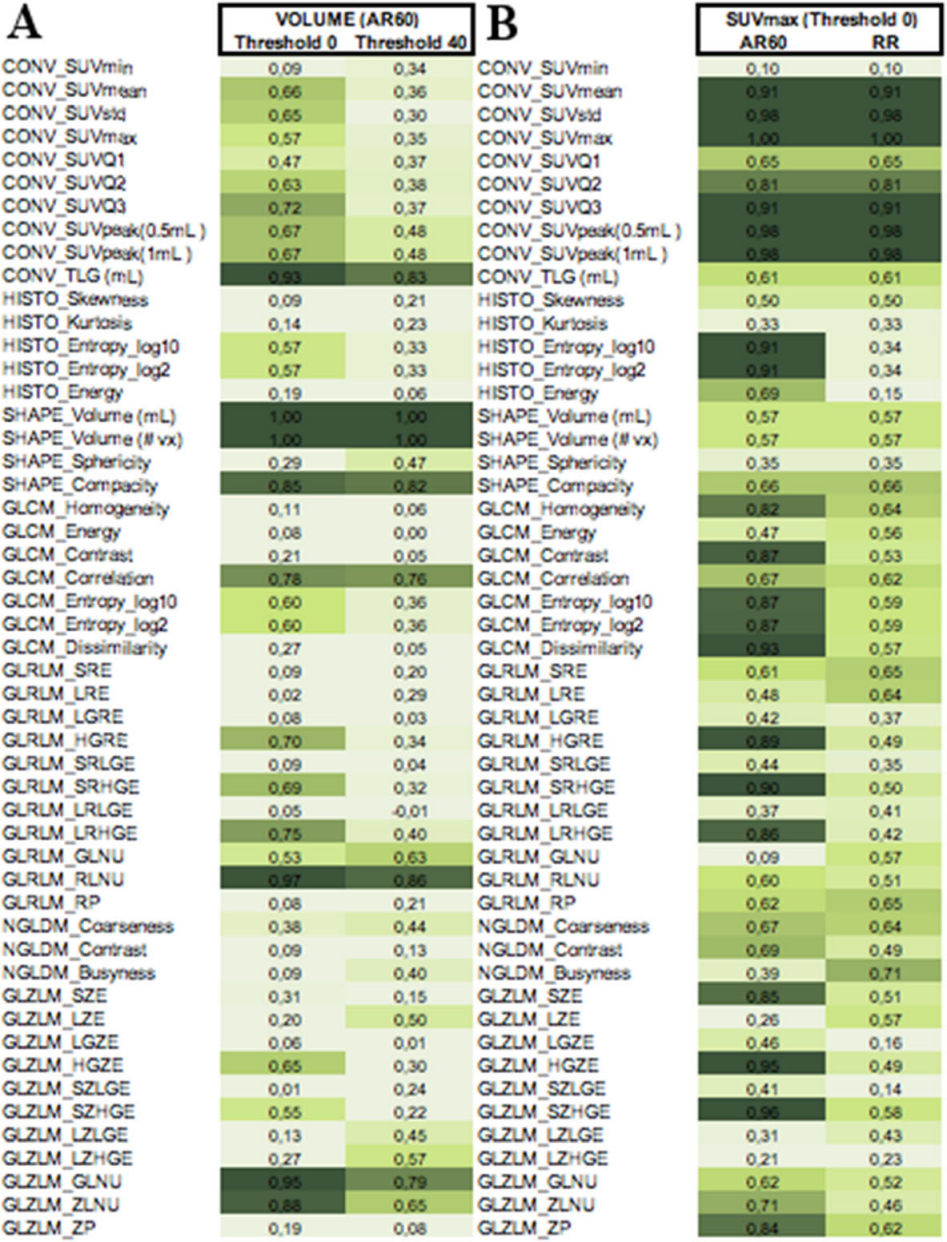
Pearson correlation coefficients (mean values between different operators) respectively (**a**) between RFs values and volume (for no threshold and 40% SUV_max_ threshold applied); and (**b**) between RFs and SUV_max_ (for AR60 and RR intensity rescale factors) without threshold. TLG (total lesion glycolysis) conventional parameter in our study corresponds to the TLSRE (total lesion somatostatin receptor expression)

Pearson correlation coefficients of RFs with volume (for AR60, without SUV_max_ threshold and with 40% SUV_max_ threshold applied, respectively) and with SUV_max_ of the ROI (AR60 and RR, no threshold applied) are shown in Fig. 7.

The following RFs showed high correlation (*r* > 0.9) with volume, regardless of SUV_max_ threshold: TLSRE, SHAPE_Compacity, GLRLM_RLNU, and GLZLM_GLNU. Regarding correlation with SUV_max_, the following RFs showed very high correlation (*r* > 0.9) when AR60 was employed: HISTO_Entropy (both_log10 and_log2), GLCM_Dissimilarity, GLRLM_SRHGE, GLZLM_HGZE, and GLZLM_SZHGE. When RR was used, no RFs were highly correlated with SUV_max_ (except for most part of conventional SUV features).

## Discussion

Our study sought to identify robust RFs in ^68^Ga-DOTA-TOC PET/CT, as a result of different segmentation and gray-level intensity discretization methods of the images. The major findings of our study were the following: (a) 64.7% of RFs (7/10 conventional, 3/6 histogram, 2/4 shape, and 21/31 textural) showed high robustness in terms of consistency (ICC > 0.9) and agreement (low median COV^*L*^ value) to different operators without SUV_max_ threshold applied (manual segmentation); (b) increasing the SUV_max_ isocontouring threshold had a positive effect on RFs robustness to segmentation. However, this approach might lead to a loss of important biological information on the population studied and reduction of analyzable lesions with textural features due to low number of voxels; (c) quantitative comparison between a semi-automatic edge-based (SAEB) algorithm and manual segmentation showed a dice similarity coefficient (DSC) of 0.75 ± 0.11 comparable to DSC between operators (0.78 ± 0.03). These results suggest that a semi-automatic algorithm might be able to aid expert segmentation to reduce operator variability and analysis time [36, 42]; (d) the use of absolute intensity rescaling factor (AR60) achieved higher robustness of RFs to segmentation than relative (RR) intensity rescaling factor.

In monocentric studies, image segmentation is one of the first step to take into account in radiomic analysis, since it is a possible source of RFs variability. Overall, RFs robustness to segmentation resulted to be quite good applying no SUV_max_ threshold (ICC > 0.9 for 64.7% RFs), using AR60 intensity discretization method (Fig. 5a). Only one morphological feature, SHAPE_Sphericity, resulted not robust to segmentation. However, its ICC value was furtherly decreased from Lifex software due to an assigned artificial feature value equal to zero for VOIs smaller than 64 voxels rather than a *Not A Number* (NAN) as other RFs. This occurred also for SHAPE_Compacity for a total of 7 lesions and only for some operator, leading to a high inter-operator variability. After removing these lesions, the ICC increased for both SHAPE_Sphericity (from ICC = 0.27 to ICC = 0.59) and SHAPE_Compacity (from ICC = 0.88 to ICC = 0.95).

In line with previous studies [52, 53], GLZLM (also called GLSZM) features and in particular the ones measuring zones with low gray-level (SZLGE and LZLGE) resulted to have a moderate robustness (ICC from 0.5 to 0.8) to segmentation (Fig. 5a). This is likely related to the lower uptake in lesions edges, where operators and SAEB segmentation showed a higher variability (Fig. 3).

When using AR60 intensity discretization method, the SUV_max_ thresholding had a considerable impact on inter-segmentation ICC values of conventional, shape, and GLRLM features (Fig. 5a). At the same time, however, it is clear that the SUV_max_ thresholding has an important impact on the dispersion (agreement) of RFs, reducing it significantly, with a progressive flattening of all RFs COV^*L*^ values toward zero (Fig. 5b, c). These results lead to considering segmentation with a 40% SUV_max_ threshold as preferable from the point of view of robustness, increasing the similarity between segmentations (Fig. 4c). However, its use may lead to a significant loss of relevant information for diagnosis and prognosis. These findings are in accordance with previous studies, reporting that segmentation with 40% SUV_max_ threshold yields superior inter-observer reproducibility of texture features in ^18^F-FDG PET/CT images, despite the loss of information about tumor heterogeneity related to exclusion of voxel intensities below the fixed 40% threshold, such as those arising from low-activity tumor regions or tumor boundaries. However, as early demonstrated by the study of Biehl et al in ^18^F-FDG PET/CT images [54], there is no consensus in the use of SUV_max_ threshold, because this parameter can lead to an over- and/or underestimation of lesion PET volume compared with CT volume related to low resolution, inherent noise, high uncertainties in lesion boundaries, and motion blurring of the lesion related to the tomography characteristics [42]. Further, from a technical point of view, the use of high SUV_max_ threshold could reduce number of voxels of the VOI denying computation of textural features. In our opinion, the concern related to the use of threshold-based segmentation methods in ^68^Ga-DOTA-peptide PET-CT images must be even higher due to the aforementioned heterogeneity of somatostatin receptors expression in neuroendocrine tumors which may explain that currently the few studies of radiomics with ^68^Ga-DOTA-peptide in the literature has been made on a manual-based segmentation [55–58].

Image gradient showed to be useful for tumor segmentation in ^18^F-FDG PET/CT images, as highlighted by Pfeahler et al. [59] and Foster et al. [42]. In this study, we demonstrated the suitability and added value of using an automatic image gradient segmentation in ^68^Ga-DOTA-peptide PET images, given the high SUV variability and the presence of high SUV values for normal tissues. For these reasons, we believe that automatic and semi-automatic segmentation methods are crucial for the radiomics analysis of NET in ^68^Ga-DOTA-peptide images.

Our results regarding tumor segmentation accuracy are promising since DSC comparing the SAEB segmentation with the manual segmentations was 0.75 ± 0.11 (median 0.77). The SAEB algorithm is characterized by its hybrid nature: the curve evolves in the image looking to both the original image (important for homogeneity) and the edge-enhanced image (important for discontinuities detection). This feature allows the SAEB algorithm to behave reproducibly within lesions in different locations, with different image appearances and different background uptakes, such as blob-like lesions with dark background (Fig. 3a, d and g), heterogeneous lesions (Fig. 3b, e, and h) and liver lesions where background present SUV values comparable to the lesion (Fig. 3c, f, and i). Thus, the use of automatic and semi-automatic segmentation methods, such as SAEB algorithm, appears to be crucial in future studies assessing robustness or clinical significance of RFs in ^68^Ga-DOTA SSTR-peptide PET/CT imaging, in order to reduce inter- and intra-reader variability of manual segmentation methods, which is also time-consuming [42, 44], and in order to reduce relevant information loss secondary to the application of 40% SUV_max_ threshold. However, further studies are needed to validate our semi-automatic segmentation methods (SAEB).

Another interesting result of our study is related to the negative impact of the relative resampling intensity discretization (which corresponded to the fixed bin number) on the robustness to segmentation of the majority of textural features analyzed. As the rescaling is carried out according to the minimum and maximum values of the VOI, the same image is rescaled differently depending on the segmentation operator/method used, leading to high variability also in RFs values. Recently, Zwanenburg et al. [20] highlighted that, as both discretization methods have their particular advantages and disadvantages, they should not be treated as equivalent. Hence, an image biomarker should be considered different to consist of the discretization method and level, in addition to its scale and base feature definition.

Anyway, the use of relative resampling is not recommended in PET images, as already observed in ^18^F-FDG PET/CT [45–47]. At least in clinical cases, the “fixed bin number” is intuitively less appropriate: it is based on the range of SUV intensities found in the volume of interest, with low SUVs corresponding to low bin numbers and high SUVs corresponding to high bin numbers; hence bin width (in SUV) and SUV range may vary between images in a cohort, even though the number of bins is consistent [20]. For the identification of RFs as new cancer-specific biomarker (e.g., NET), it is important that the textural features values would, be directly comparable, both inter- and intra-patient, in order to derive meaningful conclusions. Moreover, NETs are characterized by an extremely variable expression of somatostatin receptors in ^68^Ga-DOTA-peptides with a corresponding broader range for SUV values (from close to 0 up to higher than 100) compared to ^18^F-FDG PET/CT, causing a greater impact of the RR in the final discretized uptake values. This concept has been already emphasized by several studies: beyond all the variabilities related to the tomographs, segmentations, and post-processing settings, the robustness of RFs is also related to the tumor characteristic and behavior [52, 60–62] and to the radiotracer analyzed, as recently demonstrated by Lu et al. [63] that studied the stability of RFs for nasopharyngeal carcinoma on both ^11^C-choline and ^18^F-FDG PET/CT images, with different results. In accordance with previous studies [20, 25], the impact of intensity discretization on textural features was stronger than segmentation. This is exactly the case of textural features, where differentiation between high and low-gray levels is needed, and thus, the choice of the discretization setting is relevant. When using no SUV_max_ threshold, only four textural features resulted to be consistent: GLCM_Correlation, GLRLM_RLNU, NGLDM_Coarseness, and GLZLM_GLNU (Fig. 6).

ICC and COV^*L*^ provided in general similar information. Generally, we reported low ICC and high COV^*L*^ or the opposite. Anyway, there were also RFs with low consistency (low ICC) and high agreement (low COV^*L*^) or high consistency (high ICC) and low agreement (high COV^*L*^). Representative examples were GLCM_Entropy (both _log10 and _log2), GLRLM_SRE, GLRLM_LRE, and GLRLM_RP which had low consistency but good agreement (COV^*L*^ < 10%) to discretization. For these RFs, although measurements were not correlated when changing intensity discretization methods, the relative percentage of deviation was low (Figure S3). On the other hand, when evaluating robustness to SUV_max_ threshold, ICC was sometimes high even if COV^*L*^ was also high because, despite the great variability, the RFs remained correlated by changing the parameters, as we can observe in particular for GLRLM_LGRE, GLRLM_SRLGE, GLRLM_LRLGE, and GLZLM_LZLGE for the threshold variability (Fig. 6 and Figure S4). This behavior was due to the very small, close to zero, values of these RFs, which produced high COV^*L*^ values. Our study highlights the importance of the discretization settings chosen due to its high impact on the RFs robustness, which seems even higher in ^68^Ga-DOTA-peptide comparing to ^18^F-FDG PET/CT images (due to the widest range of SUV values in ^68^Ga-DOTA-peptide images in NET). However, discretization is a controllable variable, while segmentations do not. Respect of segmentation, there are essentially three categories of features: a first group of features almost not sensitive to segmentation with high ICC and low COV^*L*^ values (e.g. SUVmean, figure S5 a), a second group of features with high ICC with relative high COV^*L*^ values (e.g., TLG, figure S5 b) and a third group with low ICC and high COV^*L*^ values (e.g., GLZLM_SZLGE, figure S5 c), that could probably be useless in the absence of a universally standardized and totally automatic segmentation method.

Finally, regarding Pearson’s correlation between RFs and SUV_max_ and volume, most of the RFs extracted showed a poor correlation with volume. Only two textural features had a strong correlation (coefficient values > 0.8 or < − 0.8) with the volume, for all thresholds and discretization (GLRM_RLNU and GLZLM_GLNU). Regarding the correlation with SUV_max_, no features showed a strong correlation for all thresholds and discretization. These results suggest that RFs could provide additional information to better characterize NETs, regardless of volume and SUV parameters.

The extraction of robust RFs from ^68^Ga-DOTA SSTR-peptide PET/CT might contribute to solve some limitations related to the clinical evaluation of the SSTR expression in NET. At present, no consensus has been already reached regarding the assessment of patients who need to be investigated with both ^18^F-FDG and ^68^Ga-DOTA-peptide PET/CT (even if it is more probable that NET neoplasms with Ki67 > 15% will have positive lesions in ^18^F-FDG PET/CT [64, 65]). Furthermore, an early detection of more aggressive disease with ^18^F-FDG PET/CT does not necessarily reflect a change in the therapeutic strategy. Finally, conventional semi-quantitative PET parameters showed a sub-optimal feasibility to select patients for receptor radionuclide therapy (PRRT) and to evaluate response to PRRT. In this scenario, the quantitative analysis offered by radiomics might be applied as prognostic biomarker and predictor of tumor heterogeneity in NET.

### Limitation

This study is not exempt from limitations. First, we did not initially perform a phantom study. To overcome this limitation, as recommended in the radiomics quality score (RQS) proposed by Lambin et al. [66], we are currently adapting our methodology for a study on an anthropomorphic phantom filled with synthetic lesions as obtained from PET/CT images and created by the 3D printer. This model will reflect realistic tumor shapes and heterogeneity uptakes for a prospective evaluation of RFs robustness. Second, we limited our analysis to the RFs provided by the LIFEx software. Even if LIFEx’s RFs are the most representative ones, they are only a limited set of the biggest group of RFs provided by the IBSI nomenclature, furthermore, two IBSI categories are not included; further and more comprehensive studies on this aspect are needed. Third, NET are rare tumors and G3 NET and G3 NEC are rarely evaluated with ^68^Ga-DOTA SSTR-peptide; for this reason, our sample size is yet too small and heterogeneous (as shown in Table 1) to evaluate the possible correlation between robust RFs with the histological NET grading system. The phantom study will lead to the possibility to enrolled NET patients from different centers, solving also limitations regarding sample size. Fourth, the difficulty of edge-based algorithms in the segmentation of lesions with a similar uptake background or with small (< 16 cm^3^) or large dimension (> 160 cm^3^) [50] has been previously highlighted. However, we acknowledge that the number of cases analyzed in this study is not enough to quantitatively validate the algorithm in the aforementioned conditions. Nevertheless, these results encouraged us to start evaluating also this on the 3D-printer based phantom study aimed to assess the performance of SAEB analyzing both spherical lesions, non-uniform and non-spherical volumes with different sizes and different backgrounds, reproducing the human-like lesions [60, 67].

## Conclusion

Our results suggest that the use of RFs is feasible also in ^68^Ga-DOTA-TOC PET/CT. The manual delineation of VOI had an impact on RFs values dependent on RF type, preserving the correlation with high ICC values in most cases despite some relatively high COV^*L*^ values. The 40% SUV_max_ threshold increased the RFs robustness, but with a potential loss of information and analyzable lesions. Finally, the gray-level discretization influences the robustness of RFs, which vary depending on the use of relative or absolute resampling. In our opinion, an absolute resampling better suited to the evaluation of NETs with functional imaging (^68^Ga-DOTA-TOC PET/CT).

These results suggest the needing to standardize the methodology used in the radiomic PET studies in ^68^Ga-DOTA-TOC PET/CT. Moreover, a semi-automatic segmentation algorithm might be helpful to solve both the impact of different manual segmentations on RFs robustness and the loss of valuable information due to SUV_max_ threshold segmentation method.

## Supplementary Information

**Additional file 1: Table S1.** LifeX radiomic features description according to the Imaging Biomarker Standardization Initiative (IBSI) description (update 17 December 2019). LifeX version was 4.81. **Figure S1.** Box plots showing the distribution of SUVmax (panel A) and Volume (panel B). **Figure S2.** (A) Bar diagrams of intra-class correlation coefficient (ICC) values of RFs for robustness to SUVmax thresholding. Bars show the median ICC between the different segmentations for the absolute intensity rescale factor AR60. Range error bars (in black) encompass the lowest and highest values for different operators. (B) Boxplot of COVL for different threshold (20, 30, 40%) for each RFs, for the first operator (results superposable for the other operators). TLG (total lesion glycolysis) conventional parameter in our study corresponds to the TLSRE (total lesion somatostatin receptor expression). **Figure S3.** Radiomic features with moderate or poor consistency (ICC < 0.80), but high agreement (median COVL < 10%) to intensity discretization. The RFs were: GLCM_Entropy_log2, GLCM_Entropy_log10 (not shown), GLRLM_SRE, GLRLM_LRE and GLRLM_RP. Value of the RFs for each lesion are presented in the top row; boxplots of COVL for the first operator are presented in the bottom row. **Figure S4.** Radiomic features with high consistency (ICC > 0.90), but low agreement (median COV^L^ > 10%) to SUVmax thresholds (0, 20, 30 and 40%). The RFs were: GLRLM_LGRE, GLRLM_SRLGE, GLZLM_LGZE and GLZLM_LZLGE. Value of the RFs for each lesion are presented in the top row; boxplots of COV^L^ for the first operator are presented in the bottom row. **Figure S5.** Boxplot showing the distribution of RF value for each operator. The three RFs chosen are the most representative of the impact of segmentation on ICC. Segmentation did not affect SUVmean (A) and TLG (B) in terms of ICC, although TLG was characterized by not negligible dispersion (percentage of COV^L^) in our study. In contrast, segmentation had a high impact on GLZLM_SZLGE (C) in terms of both ICC and COV^L^. Mean COV^L^ of SUVmean, TLG and GLZLM_SZLGE was 8.33±3.96, 13.38±8.52 and 30.67±27.29, respectively.

## Data Availability

The datasets used and/or analyzed during the current study are available from the corresponding author on reasonable request.

## Abbreviations

^18^F-FDG: ^18^F-fluorodeoxyglucose
CT: Computed tomography
FOV: Field of view
GLCM: Gray-level co-occurrence matrix
GLNUr: Gray-level non-uniformity for run
GLNUz: Gray-level non-uniformity for zone
GLRLM: Gray-level run length matrix
GLZLM: Gray-level zone length matrix
HGRE: High gray-level run emphasis
HGZE: High gray-level zone emphasis
IBSI: Imaging biomarker standardization initiative
ICH – GCP: International Conference on Harmonization - Good Clinical Practice
LGRE: Low gray-level run emphasis
LGZE: Low gray-level zone emphasis
LRE: Long-run emphasis
LRHGE: Long-run high gray-level emphasis
LRLGE: Long-run low gray-level emphasis
LZE: Long-zone emphasis
LZHGE: Long-zone high gray-level emphasis
LZLGE: Long-zone low gray-level emphasis
MIP: Maximum intensity projection
MTV: Metabolic tumor volume
NET: Neuroendocrine tumor
NGLDM: Neighborhood gray-level different matrix
OSEM: Ordered subset expectation maximization
PET: Positron emission tomography
RF: Radiomic features
RLNU: Run length non-uniformity
ROI: Region of interest
RP: Run percentage
SAEB: Semi-automatic edge-based
SRE: Short-run emphasis
SRHGE: Short-run high gray-level emphasis
SRLGE: Short-run low gray-level emphasis
SSTR: Somatostatin receptor
SZE: Short-zone emphasis
SZHGE: Short-zone high gray-level emphasis
SZLGE: Short-zone low gray-level emphasis
SUV: Standardized uptake value
TLSRE: Total lesion somatostatin receptor expression
VOI: Volume of interest
ZLNU: Zone length non-uniformity
ZP: Zone percentage
