# Supplementary material for: Impact of segmentation and discretization on radiomic features in 68Ga-DOTA-TOC PET/CT images of neuroendocrine tumor
Source: EJNMMI Phys. 2021 Feb 27;8:21. doi: 10.1186/s40658-021-00367-6 (PMC7914329; doi:10.1186/s40658-021-00367-6)

**SUPPLEMENTAL MATERIALS**

**Table S1**. LifeX radiomic features description according to the Imaging Biomarker Standardization Initiative (IBSI) description (update 17 December 2019). LifeX version was 4.81.

| **LifeX nomenclature** | **IBSI nomenclature** | **IBSI code** | **Notes** |
| --- | --- | --- | --- |
| CONVENTIONAL_SUVmin | Minimum intensity | 1GSF |  |
| CONVENTIONAL_SUVmean | Mean intensity | Q4LE |  |
| CONVENTIONAL_SUVstd | Intensity-based mean absolute deviation | 4FUA |  |
| CONVENTIONAL_SUVmax | Maximum intensity | 84IY |  |
| CONVENTIONAL_SUVQ1 | n.d. | n.d. | Corresponding to the 25th and 75th percentile of the intensity distribution. IBSI documentation defines the 10th and 90th percentiles (QG58 and 8DWT). |
| CONVENTIONAL_SUVQ3 | n.d. | n.d. |  |
| CONVENTIONAL_SUVQ2 | Median intensity | Y12H |  |
| CONVENTIONAL_SUVpeak(1mL) | Local intensity peak | VJGA |  |
| CONVENTIONAL_SUVpeak(0.5mL) | n.d. | n.d. | Similar to local intensity peak (VJGA) but computed in a 0.5 cm3 spherical volume. |
| CONVENTIONAL_TLG (mL) | Integrated intensity | 99N0 | Total lesion glycolysis (TLG) which translated to DOTATOC PET is total lesion somatostatin receptor expression (TLSRE). |
| HISTO_Skewness | Intensity skewness | KE2A |  |
| HISTO_Kurtosis | n.d. | n.d. | In IBSI documentation, only (Excess) Intensity kurtosis (IPH6) is defined, as intensity kurtosis corrected by a Fisher correction of -3. Hence, LifeX kurtosis is equivalent apart from an offset. |
| HISTO_Entropy_log2 | Discretised intensity entropy | TLU2 |  |
| HISTO_Entropy_log10 | n.d. | n.d. | Similar to Discretised intensity entropy (TLU2) but computed using base 10. |
| HISTO_Energy | Discretised intensity uniformity | BJ5W |  |
| SHAPE_Volume (mL) | Volume (mesh) | RNU0 |  |
| SHAPE_Volume (# vx) | Volume (voxel counting) | YEKZ |  |
| SHAPE_Sphericity | Sphericity | QCFX |  |
| SHAPE_Compacity | n.d. | n.d. | In IBSI documentation, two compactness measures are defined: Compactness 1 (SKGS) and Compactness 2 (BQWJ) which slightly differ from the LifeX compacity, i.e.:  F_morph.comp.1_ = 1/(π^(1/2) ∙ SHAPE_Compacity)  F_morph.comp.2_ =((36π)^(1/2))/SHAPE_Compacity |
| GLCM_Homogeneity | Inverse difference | IB1Z |  |
| GLCM_Energy | Angular second moment | 8ZQL |  |
| GLCM_Contrast | Contrast | ACUI |  |
| GLCM_Correlation | Correlation | NI2N |  |
| GLCM_Entropy_log2 | Joint entropy | TU9B |  |
| GLCM_Entropy_log10 | n.d. | n.d. | Similar to Joint entropy (TU9B) but computed using base 10. |
| GLCM_Dissimilarity | Dissimilarity | 8S9J |  |
| GLRLM_SRE | Short runs emphasis | 220V |  |
| GLRLM_LRE | Long runs emphasis | W4KF |  |
| GLRLM_LGRE | Low grey level run emphasis | V3SW |  |
| GLRLM_HGRE | High grey level run emphasis | G3QZ |  |
| GLRLM_SRLGE | Short run low grey level emphasis | HTZT |  |
| GLRLM_SRHGE | Short run high grey level emphasis | GD3A |  |
| GLRLM_LRLGE | Long run low grey level emphasis | IVP0 |  |
| GLRLM_LRHGE | Long run high grey level emphasis | 3KUM |  |
| GLRLM_GLNUr | Grey level non-uniformity | R5YN |  |
| GLRLM_RLNU | Run length non-uniformity | W93Y |  |
| GLRLM_RP | Run percentage | 9ZK5 |  |
| NGLDM_Coarseness | Coarseness | QCDE |  |
| NGLDM_Contrast | Contrast | 65HE |  |
| NGLDM_Busyness | Busyness | NQ30 |  |
| GLZLM_SZE | Small zone emphasis | 5QRC |  |
| GLZLM_LZE | Large zone emphasis | 48P8 |  |
| GLZLM_LGZE | Low grey level zone emphasis | XMSY |  |
| GLZLM_HGZE | High grey level zone emphasis | 5GN9 |  |
| GLZLM_SZLGE | Small zone low grey level emphasis | 5RAI |  |
| GLZLM_SZHGE | Small zone high grey level emphasis | HW1V |  |
| GLZLM_LZLGE | Large zone low grey level emphasis | YH51 |  |
| GLZLM_LZHGE | Large zone high grey level emphasis | J17V |  |
| GLZLM_GLNUz | Grey level non-uniformity | JNSA |  |
| GLZLM_ZLNU | Zone size non-uniformity | 4JP3 |  |
| GLZLM_ZP | Zone percentage | P30P |  |

**Figure S1.** Box plots showing the distribution of SUVmax (panel A) and Volume (panel B).


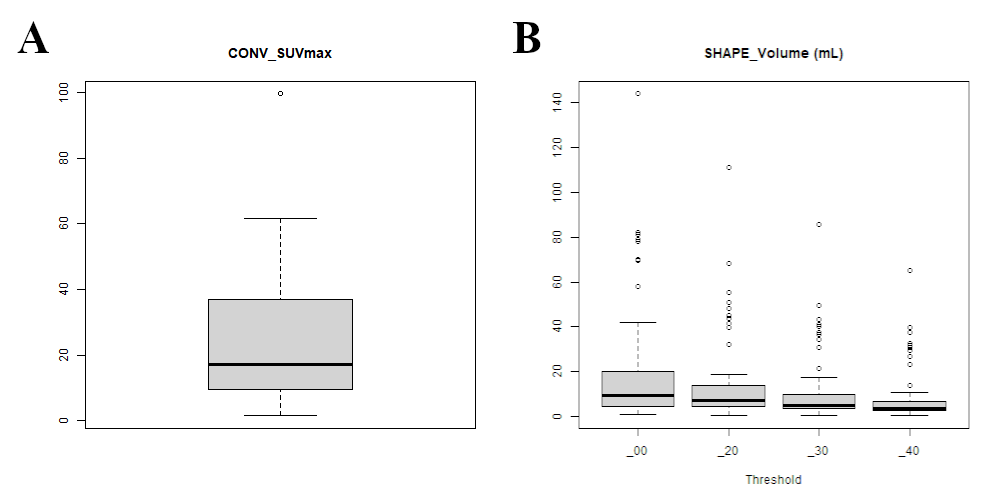


**Figure S2.** **(**A) Bar diagrams of intra-class correlation coefficient (ICC) values of RFs for robustness to SUV_max_ thresholding. Bars show the median ICC between the different segmentations for the absolute intensity rescale factor AR60. Range error bars (in black) encompass the lowest and highest values for different operators. (B) Boxplot of COV^L^ for different threshold (20, 30, 40%) for each RFs, for the first operator (results superposable for the other operators). TLG (total lesion glycolysis) conventional parameter in our study corresponds to the TLSRE (total lesion somatostatin receptor expression).

**
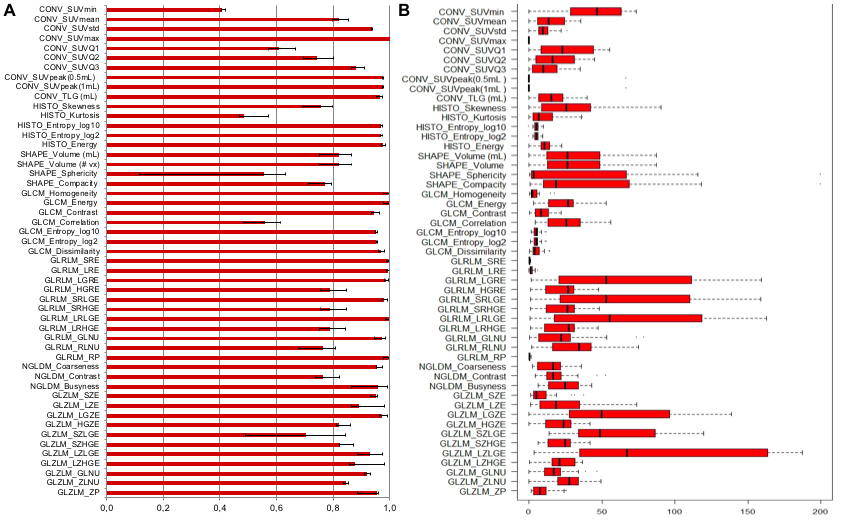
**

**Figure S3**. Radiomic features with moderate or poor consistency (ICC < 0.80), but high agreement (median COV^L^ < 10%) to intensity discretization. The RFs were: GLCM_Entropy_log2, GLCM_Entropy_log10 (not shown), GLRLM_SRE, GLRLM_LRE and GLRLM_RP. Value of the RFs for each lesion are presented in the top row; boxplots of COV^L^ for the first operator are presented in the bottom row.


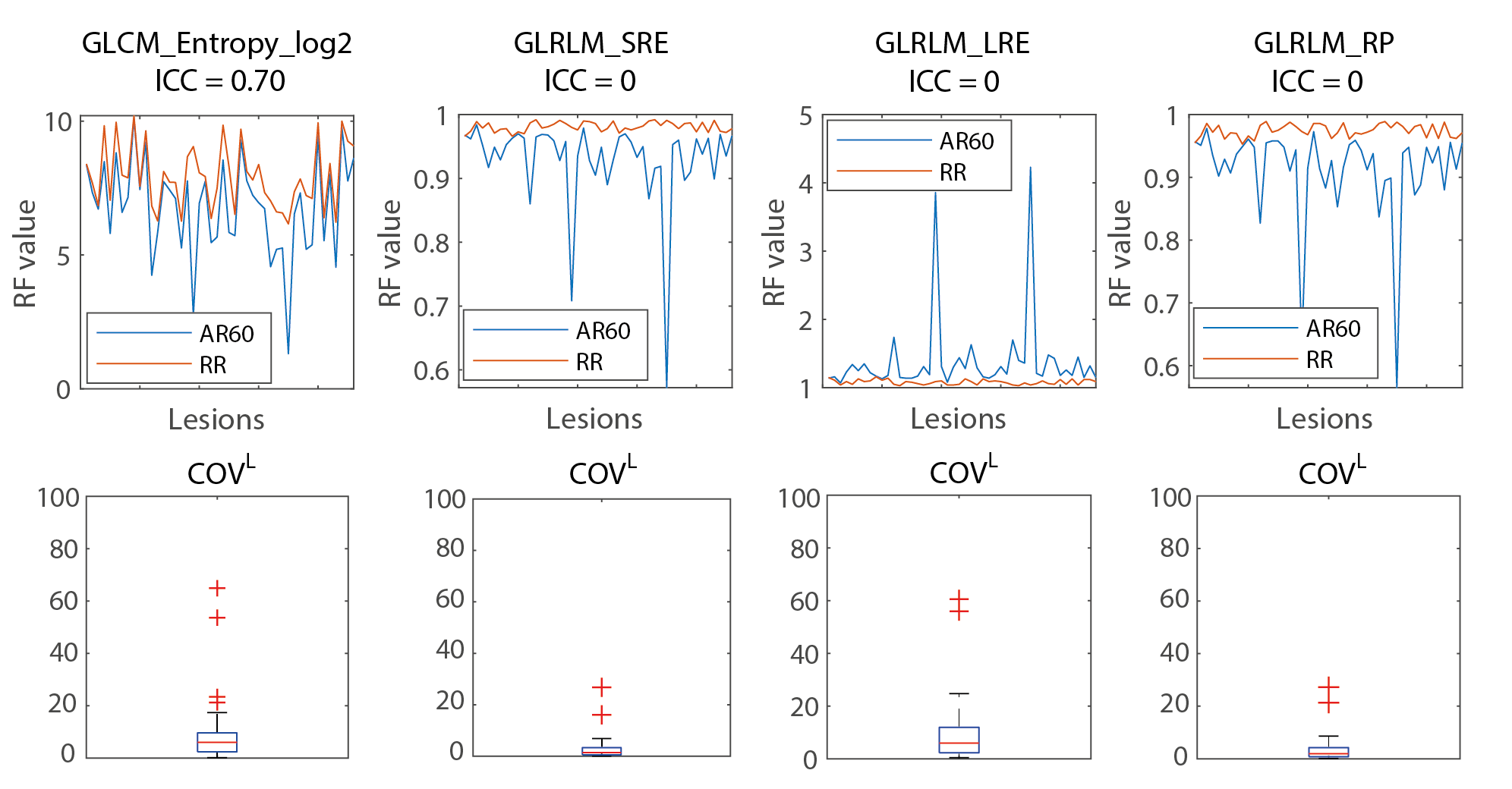


**Figure S4**. Radiomic features with high consistency (ICC > 0.90), but low agreement (median COV^L^ > 10%) to SUVmax thresholds (0, 20, 30 and 40%). The RFs were: GLRLM_LGRE, GLRLM_SRLGE, GLZLM_LGZE and GLZLM_LZLGE. Value of the RFs for each lesion are presented in the top row; boxplots of COV^L^ for the first operator are presented in the bottom row.


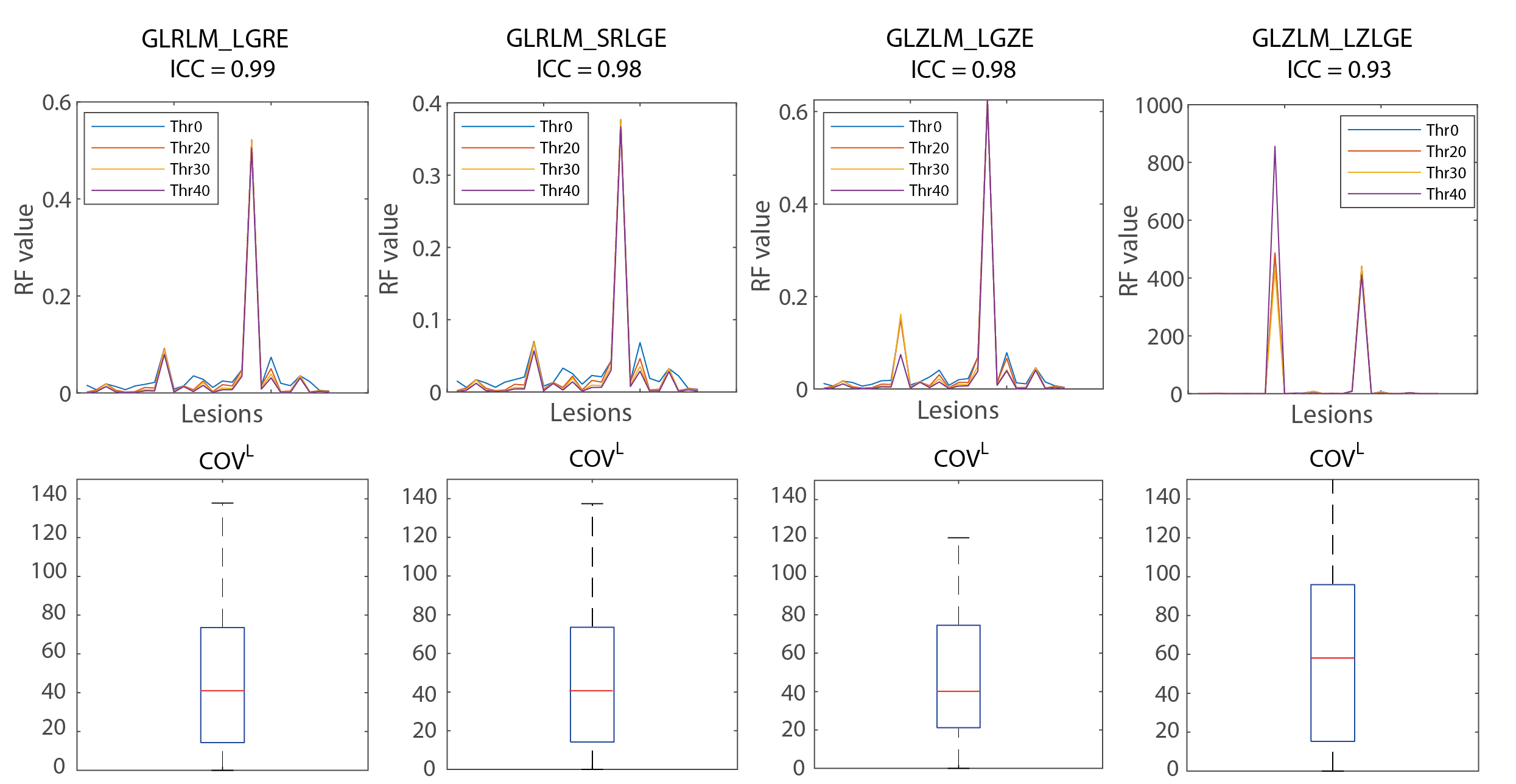


**Figure S5**. Boxplot showing the distribution of RF value for each operator. The three RFs chosen are the most representative of the impact of segmentation on ICC. Segmentation did not affect SUVmean (A) and TLG (B) in terms of ICC, although TLG was characterized by not negligible dispersion (percentage of COV^L^) in our study. In contrast, segmentation had a high impact on GLZLM_SZLGE (C) in terms of both ICC and COV^L^. Mean COV^L^ of SUVmean, TLG and GLZLM_SZLGE was 8.33±3.96, 13.38±8.52 and 30.67±27.29, respectively.


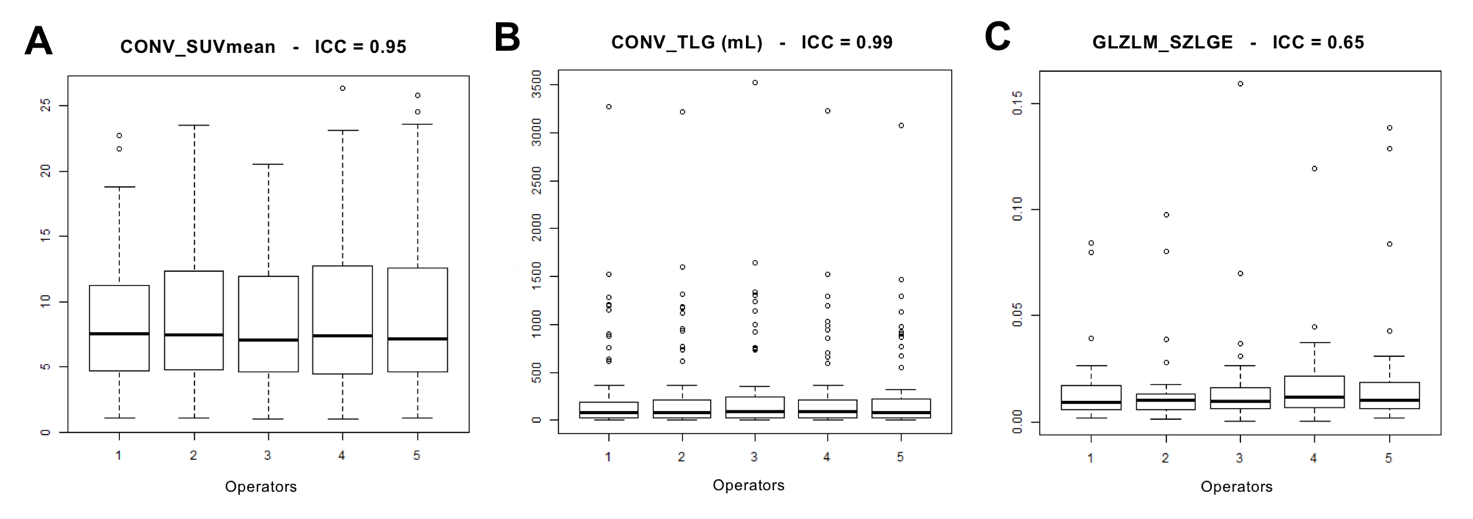

Supplement: Supplementary file 1 — Additional file 1: Table S1. LifeX radiomic features description according to the Imaging Biomarker Standardization Initiative (IBSI) description (update 17 December 2019). LifeX version was 4.81. Figure S1. Box plots showing the distribution of SUVmax (panel A) and Volume (panel B). Figure S2. (A) Bar diagrams of intra-class correlation coefficient (ICC) values of RFs for robustness to SUVmax thresholding. Bars show the median ICC between the different segmentations for the absolute intensity rescale factor AR60. Range error bars (in black) encompass the lowest and highest values for different operators. (B) Boxplot of COVL for different threshold (20, 30, 40%) for each RFs, for the first operator (results superposable for the other operators). TLG (total lesion glycolysis) conventional parameter in our study corresponds to the TLSRE (total lesion somatostatin receptor expression). Figure S3. Radiomic features with moderate or poor consistency (ICC < 0.80), but high agreement (median COVL < 10%) to intensity discretization. The RFs were: GLCM_Entropy_log2, GLCM_Entropy_log10 (not shown), GLRLM_SRE, GLRLM_LRE and GLRLM_RP. Value of the RFs for each lesion are presented in the top row; boxplots of COVL for the first operator are presented in the bottom row. Figure S4. Radiomic features with high consistency (ICC > 0.90), but low agreement (median COVL > 10%) to SUVmax thresholds (0, 20, 30 and 40%). The RFs were: GLRLM_LGRE, GLRLM_SRLGE, GLZLM_LGZE and GLZLM_LZLGE. Value of the RFs for each lesion are presented in the top row; boxplots of COVL for the first operator are presented in the bottom row. Figure S5. Boxplot showing the distribution of RF value for each operator. The three RFs chosen are the most representative of the impact of segmentation on ICC. Segmentation did not affect SUVmean (A) and TLG (B) in terms of ICC, although TLG was characterized by not negligible dispersion (percentage of COVL) in our study. In contrast, segmentation had a high impact on GL [file 40658_2021_367_MOESM1_ESM.docx]
